# CCR1^+^ monocytes facilitating bronchopulmonary dysplasia through regulation of S100A8 and MMP8

**DOI:** 10.3389/fimmu.2026.1809178

**Published:** 2026-05-01

**Authors:** Yingjuan Geng, Qing Chen, Menghao Wang, Shasha Wang, Yixian Ji, Xingyu Ji, Mingming Lu, Aifeng Wang, Haiyan Zhu, Jian Hu

**Affiliations:** 1 Department of Pediatrics, The Affiliated Huaian No. 1 People’s Hospital of Nanjing Medical University, Huai’an, China; 2 Department of Pediatrics, Huai’an Clinical Medical College of Jiangsu University, Huai’an, China; 3 Department of Pediatrics, Huai’an Clinical College of Xuzhou Medical University, Huai’an, China

**Keywords:** bronchopulmonary dysplasia, inflammation, monocytes, myeloid cells, single-cell transcriptome

## Abstract

Bronchopulmonary dysplasia (BPD) is the most common chronic lung disease in preterm infants and is characterized by aberrant late-stage lung development driven by multiple factors. Although previous studies have demonstrated that BPD is associated with prenatal and postnatal inflammatory stimuli, the inflammatory mechanisms involved immune cell populations have not been fully elucidated. Here, we systematically investigated the roles of myeloid cells in the inflammatory processes underlying BPD. Using single-cell transcriptomic data of normal and BPD mouse lung, we found that the proportions of several myeloid subpopulations were specifically upregulated in BPD lung, including CCR1^+^ monocytes, CD74^+^ macrophages, IL7R^+^ macrophages, MARCO^+^ macrophages, CD63^+^ neutrophils, and CD73^+^ neutrophils. However, we only validated the increased proportions of CCR1^+^ monocytes in human BPD blood through flow cytometry analysis. Combined with bulk transcriptome and detection in serum of human BPD blood, we identified S100A8 and MMP8 as highly expressed downstream factors of CCR1^+^ monocytes. Immunofluorescence staining further confirmed the presence of CCR1^+^ monocytes expressing S100A8 or MMP8 in the mouse BPD lung tissues and demonstrated their association with disease development. Finally, in CCR1^+^ monocytes isolated from the peripheral blood of BPD infants, we found that the NF-κB and AKT pathways regulate the production of S100A8 and MMP8 respectively. Our study explored the role and mechanism of CCR1^+^ monocytes associated with BPD, which offers valuable insights for the development of novel clinical biomarkers and therapeutic strategies for BPD.

## Introduction

Bronchopulmonary dysplasia (BPD) is the most common complication in preterm infants, and its incidence has shown an increasing trend in recent years (1). BPD can occur at different stages of lung development and is caused by multifactorial injurious stimuli, including genetic and environmental factors. Histopathologically, BPD is characterized by arrested development, remodeling, and functional impairment of the airways, alveoli, mesenchyme, and pulmonary vasculature (2, 3). BPD is not merely a pulmonary disorder but a systemic disease with lifelong consequences for health and quality of life, affecting up to 45% of infants born at a gestational age of 29 weeks (4). Preterm infants with moderate to severe BPD are at a substantially increased risk of developing pulmonary hypertension, which contributes to elevated mortality rates (5, 6).

Inflammation represents a common pathway leading to lung injury and progression to BPD. Oxygen supplementation, positive-pressure ventilation, and postnatal sepsis can all induce pulmonary inflammation and are clinically associated with the development of BPD. In preclinical animal models, exposure to hyperoxia or positive-pressure ventilation alone is sufficient to induce BPD-like phenotypes, including impaired alveolarization and microvascular injury. Numerous studies have demonstrated that blockade of inflammatory mediators, receptors, and signaling pathways can ameliorate BPD phenotypes in animal models (7, 8). In the early stages of lung injury in preterm infants with BPD, elevated levels of IL-8 and IL-6 are accompanied by increased inflammatory cells in tracheal aspirates, such as pro-inflammatory CD4^+^ T cells and myeloid cells. These changes initiate a cascade of deleterious inflammatory responses, leading to pulmonary microvascular edema, restricted expression of epithelial growth factors, and inhibition of lung development (9, 10). Several myeloid cell types, including macrophages, neutrophils, and monocytes, have been reported to be recruited to the lungs during BPD development, where they drive local inflammatory responses and contribute to impaired alveolarization (11–13).

The advent of single-cell RNA sequencing (scRNA-seq) has greatly enhanced the resolution of tissue microenvironment analysis, enabling in-depth characterization of individual cells and cellular subpopulations. Previous studies using scRNA-seq have analyzed mesenchymal stromal cells (MSCs) in lung tissues from hyperoxia-induced BPD mouse models and demonstrated a marked expansion of Ly6a^+^ MSCs in BPD lungs, accompanied by upregulation of multiple pro-inflammatory, pro-fibrotic, and anti-angiogenic factors. Among these, the Ly6a^+^Col14a1^+^ MSC subpopulation was identified as being closely associated with lung development (14). In addition to MSCs, epithelial cells, endothelial cells, lymphocytes, and myeloid cells have also been shown to undergo significant alterations in cellular differentiation and transcriptional profiles in hyperoxia-injured mouse lungs. Intercellular communication analyses further indicate that inflammatory signaling serves as a major driving force in BPD pathogenesis (15). However, comprehensive characterization of myeloid cell subpopulations and their functional roles during BPD development remains limited. In this study, we integrated lung tissue single-cell transcriptomic data with peripheral blood transcriptomic analyses to investigate the diversity of myeloid cell differentiation and its association with BPD. Furthermore, we validated the roles and molecular mechanisms of BPD-associated monocyte subpopulations using lung tissue sections and cell experiment *in vitro*.

## Materials and methods

### Flow cytometry

Fresh blood samples from healthy and BPD preterm infants were obtained from the Affiliated Huaian No. 1 People’s Hospital of Nanjing Medical University. Single-cell suspensions were treated with erythrocyte lysis buffer. Cells were stained with directly conjugated antibodies for 45 min at 4 °C in the dark in PBS including 1% BSA, washed with buffer and analyzed using a flow cytometer (Fortessa, BD). The following antibodies were purchased from BD: CD45 (747338), CD11b (562632), CD14 (555397), CD68 (562117), IL7R (561028), CD66b (555724), CD63 (561925), and CD73 (561014). The following antibodies were purchased from R&D Systems: CCR1 (FAB145P-025). The following antibodies were purchased from BioLegend: CD74 (326807). The following antibodies were purchased from abcam: MARCO (ab319451). Cell populations were identified based on cell marker expression as previously described. The resulting data were analyzed using FlowJo Software (v10.5.3).

### Isolation of CCR1^+^ monocytes

Fresh blood samples from BPD preterm infants were obtained from the Affiliated Huaian No. 1 People’s Hospital of Nanjing Medical University. The serum was collected by centrifugation and was used for subsequent cell culture. Single-cell suspensions were treated with erythrocyte lysis buffer. Cells were stained and washed as above. Human CCR1^+^ monocytes were sorted by FACS based on cell surface marker expression (CD45^+^CD11b^+^CD14^+^CCR1^+^) in single-cell suspensions. The collected monocytes were subjected to the following treatments respectively: no serum (only RPMI 1640 medium), serum, serum with MAPK inhibitor SCH772984 (HY-50846, MCE), serum with NF-κB inhibitor BAY 11-7082 (HY-13453, MCE), serum with AKT inhibitor MK-2206 (2HCl) (HY-10358, MCE). After incubation for 6 hours, the cell supernatant was collected by centrifugation. The following ELISA kits were used to detect the protein levels in the cell supernatant: Human MMP8 ELISA kit (KE00329, proteintech), Human S100A8 ELISA kit (EHS100A8, Thermo Fisher). The MMP8 and S100A8 protein levels in serum from healthy and BPD preterm infants were also detected as above.

### Animal experiment and immunofluorescence staining

Pregnant C57BL/6 mice were purchased from Charles Rivers Laboratories, Beijing, China. Newborn mice were randomized at day of birth and divided to two groups, which were either maintained in room air (normoxia, 21% O_2_) or in normobaric hyperoxia (85% O_2_). All the mice were sacrificed after 14 days. The lung tissues of mice were used for immunofluorescence staining. All animal procedures were approved by the Animal Care Committee of the Affiliated Huaian No. 1 People’s Hospital of Nanjing Medical University under the approval number DW-P-2024-025-01. Fresh mouse lung tissue samples were embedded in OCT compound (4583, Solarbio) and frozen at -80 °C. Cryosections were prepared using a cryostat and incubated at room temperature with blocking/permeabilization solution for 30 minutes, followed by overnight incubation at 4 °C with primary antibodies, including anti-S100A8 antibody (1:200, ab92331, Abcam), anti-MMP8 antibody (1:200, 17874-1-AP, Proteintech), anti-CCR1 antibody (1:200, 152502, BioLegend), anti-LY6C antibody (1:200, sc-271811, Santa Cruz). After primary incubation, sections were incubated with secondary antibodies at room temperature for 2 hours, including Donkey Anti-Rat IgG (H+L) conjugated Alexa Fluor 488 (1:1000, 712-545-150, Jackson ImmunoResearch), Donkey Anti-Mouse IgG (H+L) conjugated Alexa Fluor 555 (1:1000, 715-565-150, Jackson ImmunoResearch), and Donkey Anti-Rabbit IgG (H+L) conjugated Alexa Fluor 647 (1:1000, 711-605-152, Jackson ImmunoResearch), followed by incubation with DAPI (1:50,000, 10236276001, Roche) for 15 minutes. Images were captured using a confocal microscope.

### Single-cell RNA sequencing data analysis

The murine BPD lung tissue dataset used in this study was obtained from the Gene Expression Omnibus (GEO) database under accession number GSE151974 (15). The scRNA-seq data in GSE151974 were generated using the MULTI-seq approach and comprised approximately 66,000 cells derived from normal and hyperoxia-injured lung tissues of 36 mice. All single-cell raw data were obtained from previously published studies. Single-cell data analyses were performed in R (version 4.1.0) using the Seurat package (16, 17). The complete analysis workflow included the following steps: construction of the Seurat object, standard pre-processing, data normalization, identification of highly variable features, data scaling, linear dimensional reduction, determination of dataset dimensionality, cell clustering, non-linear dimensional reduction (UMAP/t-SNE), identification of differentially expressed features (cluster markers), assignment of cell type identities to clusters, and data visualization. All scRNA-seq count matrices analyzed in this study were downloaded directly from GEO. Prior to data integration, each dataset was processed individually, including quality control and cell filtering, data normalization, and identification of highly variable genes. Dataset integration was subsequently performed using the Seurat integration workflow to better characterize the molecular mechanisms underlying BPD pathogenesis and differences in cellular composition. Cross-dataset cell anchors corresponding to matched biological states were identified, and technical batch effects between datasets were corrected. Datasets from different time points were integrated for downstream analyses. The integrated dataset was then subjected to standard Seurat processing, including data scaling, linear dimensional reduction using principal component analysis (PCA), non-linear dimensional reduction using uniform manifold approximation and projection (UMAP), and cell clustering.

### Visualization of single-cell transcriptomic data

Visualization of single-cell data analyses was also conducted using Seurat, including UMAP scatter plots, violin plots, dot plots, volcano plots, and heatmaps. UMAP scatter plots display the spatial distribution of individual cells based on gene expression profiles, with cells exhibiting similar expression patterns located in close proximity. Violin plots illustrate the expression levels of specific genes or gene sets within defined cell populations. In dot plots, color intensity represents the average expression level of a given gene within a cell type, with darker colors indicating higher expression, while dot size reflects the proportion of cells with positive expression, with larger dots indicating higher positivity. Volcano plots depict differentially expressed genes between a given cell population and other cell types, including both upregulated and downregulated genes. Heatmaps compare the average expression levels of selected genes across different cell types.

### RNA sequencing data analysis

Bulk transcriptomic data from peripheral blood cells of preterm infants with BPD were obtained from the GEO database under accession number GSE220135 (18). This dataset includes samples from non-BPD and BPD preterm infants, with peripheral blood cells collected at three time points: cord blood, postnatal day 14, and postnatal day 28. Data normalization and differential expression analyses were performed in R using the limma package. Based on differential gene expression analyses of distinct myeloid cell subpopulations identified in the single-cell transcriptomic data, sets of subpopulation-specific upregulated genes were extracted. These gene signatures were subsequently used to perform gene set variation analysis (GSVA) on the GSE220135 bulk transcriptomic dataset to estimate the relative abundance scores of these myeloid cell subpopulations in peripheral blood samples from preterm infants (19).

### Gene enrichment analysis

Differentially expressed genes for each cluster were identified using Seurat. Lists of upregulated or downregulated genes were uploaded to the Database for Annotation, Visualization and Integrated Discovery (DAVID) online tool (https://davidbioinformatics.nih.gov/), which provides a comprehensive suite of functional annotation tools to facilitate the biological interpretation of large gene lists (20). Gene ontology (GO) biological process enrichment analysis was performed to identify significantly enriched functional categories associated with each gene set.

### Statistical analysis

Data analysis was performed using GraphPad Prism (version 8). An unpaired t-test was used for comparisons between two groups. Ordinary one-way ANOVA was used for comparisons between multiple groups. Tukey’s multiple comparisons test was used for *post hoc* test after one-way ANOVA. Each group consists of three repetitions. Single-cell RNA-seq analyses were conducted using R software (version 4.1.0). Statistical significance is indicated in the figure legends. Data are presented as mean ± SEM, with significance thresholds set as P < 0.05 (*P < 0.05, **P < 0.01, ***P < 0.001, ****P < 0.0001, ns, not significant).

## Results

### Single-cell RNA-seq analysis of lungs from normoxic and hyperoxia-injured mice

**Figure 1:**
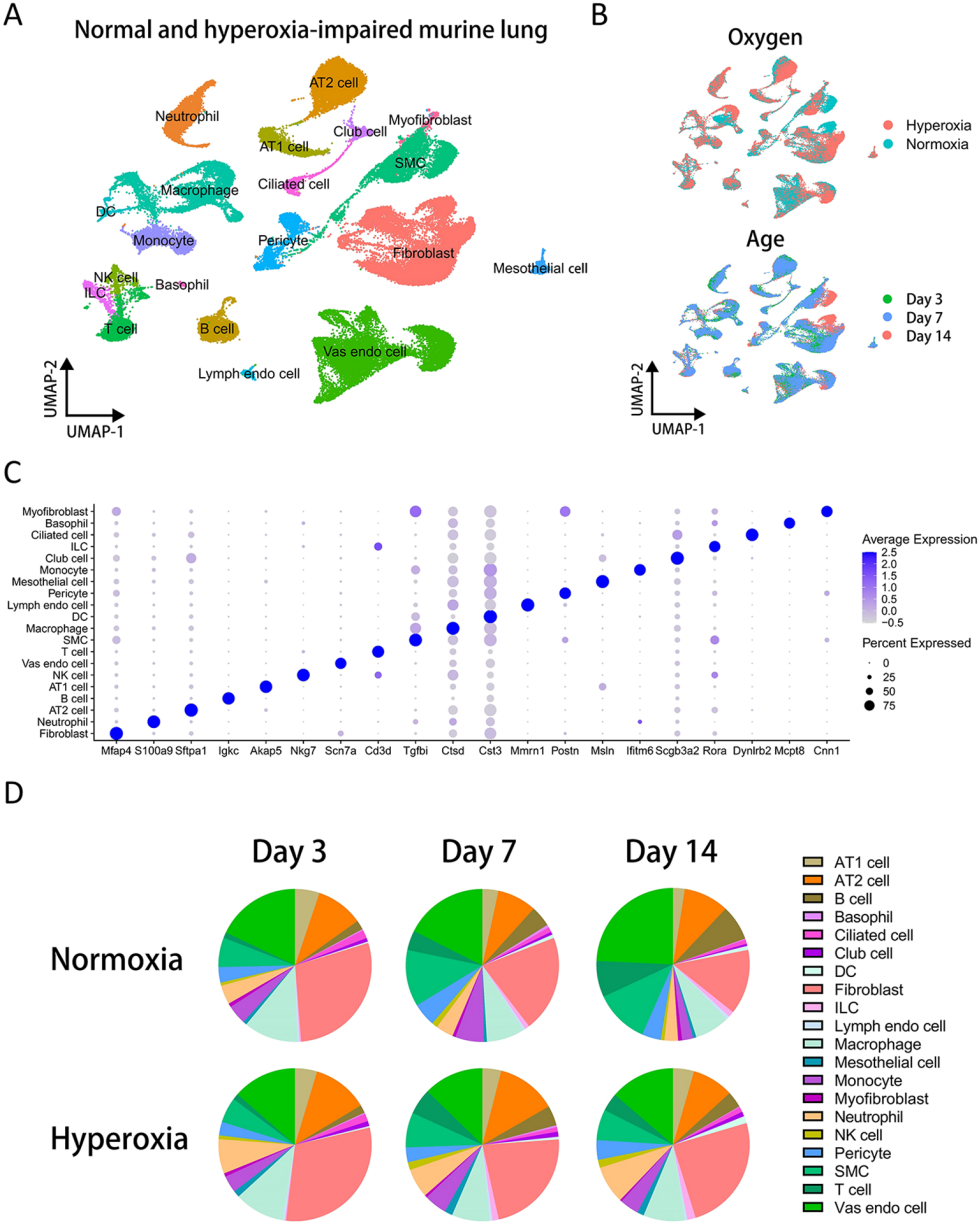
Sc-RNAseq analysis of normal and hyperoxia-impaired murine lung. **(A)** UMAP plot displaying cell clustering of normal and hyperoxia-impaired murine lung. **(B)** UMAP plots colored by different condition of oxygen or age of mouse. **(C)** Dot plot showing representative marker genes of all cell types. **(D)** Pie plots comparing proportions of all cell types in normoxia and hyperoxia groups on day 3, day 7, or day 14.

To investigate the mechanisms and molecular basis underlying bronchopulmonary dysplasia (BPD) in preterm infants, we collected single-cell RNA sequencing (scRNA-seq) data of lung tissues from neonatal mouse models of hyperoxia-induced lung injury from the NCBI Gene Expression Omnibus (GEO) database. The dataset comprised lung tissues from mice exposed to normoxia or hyperoxia at different postnatal time points. Based on previously reported marker genes, we identified 20 distinct cell types: fibroblasts (Mfap4), neutrophils (S100a9), alveolar type II (AT2) cells (Sftpa1), B cells (Igkc), alveolar type I (AT1) cells (Akap5), NK cells (Nkg7), vascular endothelial cells (Scn7a), T cells (Cd3d), smooth muscle cells (Tgfbi), macrophages (Ctsd), DCs (Cst3), lymphatic endothelial cells (Mmrn1), pericytes (Postn), mesothelial cells (Msln), monocytes (Ifitm6), club cells (Scgb3a2), innate lymphoid cells (Rora), ciliated cells (Dynlrb2), basophils (Mcpt8), and myofibroblasts (Cnn1). Following dimensionality reduction, all cells were visualized using UMAP plot, and each cell cluster was annotated with its corresponding cell-type identity (**Figure 1A**). To evaluate data integration, UMAP plots were used to display the distribution of cells across treatment conditions (normoxia and hyperoxia) as well as across postnatal time points (Day 3, Day 7, and Day 14). Cells from different groups were evenly distributed within the UMAP space, indicating that data integration effectively minimized batch effects and technical noise (**Figure 1B**). A heatmap of differentially expressed genes demonstrated that each cell type exhibited significant upregulation of its characteristic gene signatures (**Supplementary Figure 1**). Additionally, dot plots illustrating representative marker genes showed highly specific expression patterns corresponding to each annotated cell type (**Figure 1C**).

To assess changes in cellular composition under normoxic and hyperoxic conditions at different developmental stages, we compared cell-type proportions at Day 3, Day 7, and Day 14. Pie charts revealed that fibroblasts, vascular endothelial cells, and macrophages constituted the largest proportions across all groups. However, no significant differences were observed in the proportions of these major cell populations between normoxia and hyperoxia at any time point. In contrast, neutrophils exhibited the most pronounced changes in cell proportion, showing a consistently higher abundance in the hyperoxia group across all three time points (**Figure 1D**). This finding suggests a potential association between neutrophils and hyperoxia-induced stress responses, possibly contributing to lung injury and inflammation. Nevertheless, the absence of significant changes at the total cell-type level does not exclude the involvement of specific subpopulations within other cell types. To further elucidate the association between inflammation and myeloid cells in BPD pathogenesis, we next focused on the heterogeneity and differentiation of myeloid cell subpopulations before and after hyperoxic injury.

### Differential analysis of myeloid cell subpopulations

**Figure 2:**
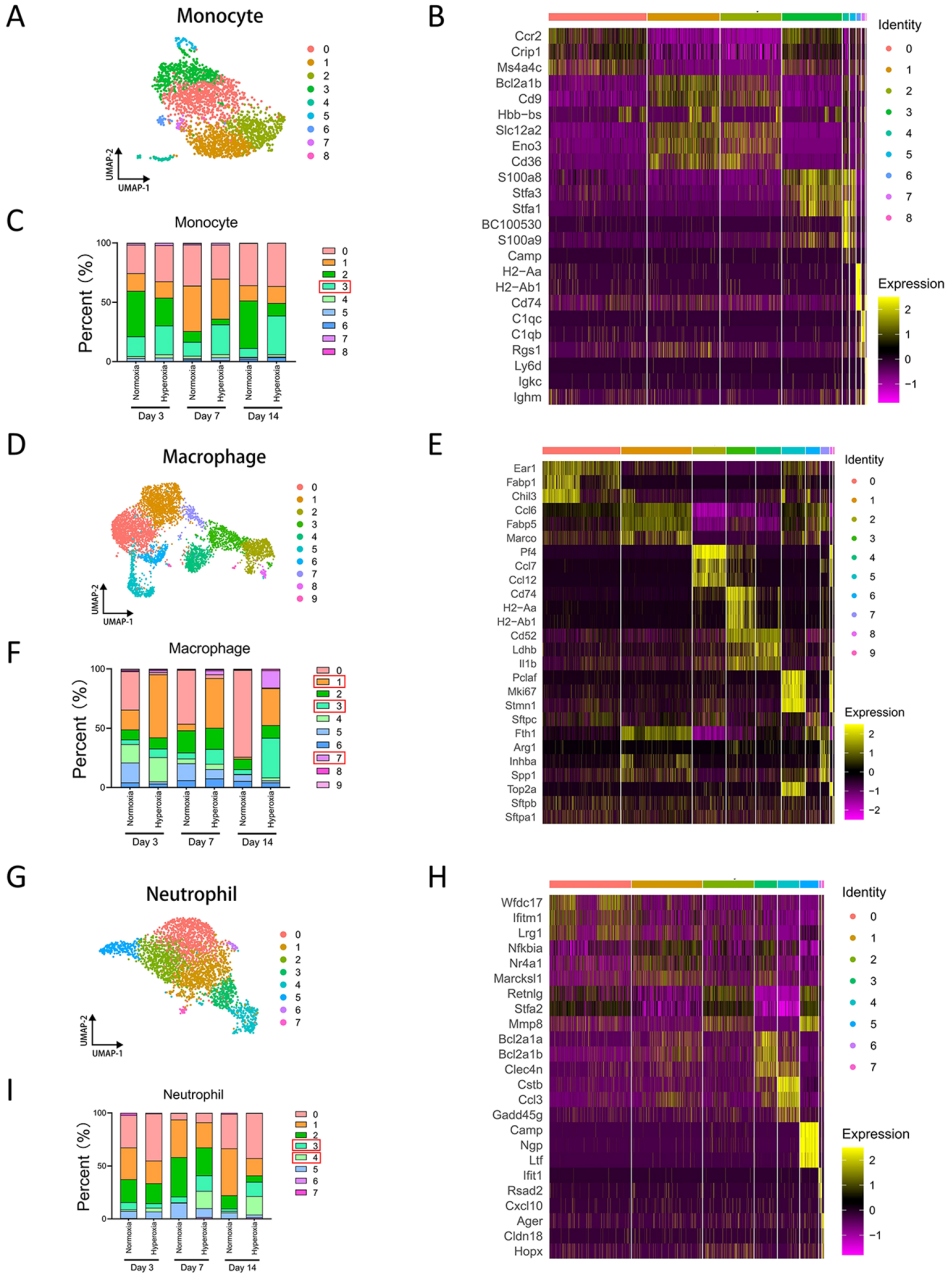
Sc-RNAseq analysis of monocytes, macrophages, and neutrophils. **(A)** UMAP plot displaying cell clustering of monocytes. **(B)** Heatmap showing representative marker genes of all monocyte clusters. **(C)** Histogram comparing proportions of all monocyte clusters in normoxia and hyperoxia groups on day 3, day 7, or day 14. **(D)** UMAP plot displaying cell clustering of macrophages. **(E)** Heatmap showing representative marker genes of all macrophage clusters. **(F)** Histogram comparing proportions of all macrophage clusters in normoxia and hyperoxia groups on day 3, day 7, or day 14. **(G)** UMAP plot displaying cell clustering of neutrophils. **(H)** Heatmap showing representative marker genes of all neutrophil clusters. **(I)** Histogram comparing proportions of all neutrophil clusters in normoxia and hyperoxia groups on day 3, day 7, or day 14.

We extracted four major myeloid cell populations for reanalysis at higher resolution. Monocytes were subdivided into 9 distinct clusters following dimensionality reduction, as visualized by UMAP (**Figure 2A**). Analysis of mitochondrial and ribosomal gene expression revealed low mitochondrial gene proportions across all nine clusters, indicating high cellular viability and minimal apoptosis (**Supplementary Figure 2A**). Differential gene expression analysis across the nine monocyte clusters was showed in heatmap (**Figure 2B**). To examine differences in monocyte cluster composition across experimental groups, we analyzed normoxia and hyperoxia conditions at Day 3, Day 7, and Day 14 (**Supplementary Figure 4**). Quantitative analysis using bar plots revealed that monocyte cluster 3 exhibited a consistent and progressive increase in the hyperoxia group at all time points, with the magnitude of enrichment positively correlated with postnatal age. These findings indicate that monocyte cluster 3 is selectively expanded in hyperoxia-injured lungs and may play a critical role in hyperoxia-induced pathological processes (**Figure 2C**).

Macrophages were further subdivided into 10 distinct clusters (**Figure 2D**). Violin plots of mitochondrial and ribosomal gene expression showed mitochondrial gene proportions below 10% across all clusters, consistent with non-apoptotic cells. Ribosomal gene expression varied modestly among clusters, suggesting differences in transcriptional activity and protein synthesis capacity (**Supplementary Figure 2B**). Differential gene expression analysis revealed greater transcriptional heterogeneity among macrophage clusters compared with monocytes, with particularly pronounced differences observed in clusters 2 and 3 (**Figure 2E**). Comparative clustering analyses between normoxia and hyperoxia groups demonstrated striking differences in macrophage cluster distributions. Cluster 0 was predominantly present in normoxic lungs and was nearly depleted following hyperoxic exposure, whereas cluster 1 showed the opposite pattern, being markedly enriched under hyperoxic conditions at all time points (**Supplementary Figure 4**). Quantitative analysis further revealed that cluster 5 also declined substantially after hyperoxic injury. In contrast, clusters 1, 3, and 7 were significantly increased in hyperoxia-injured lungs, with enrichment becoming more pronounced with increasing postnatal age. These findings suggest that macrophage cluster 0 and 5 may be associated with normal lung homeostasis, whereas cluster 1, 3, and 7 likely represent stress-responsive macrophages that contribute to or exacerbate hyperoxia-induced lung injury and impaired bronchial development (**Figure 2F**).

Neutrophils were reclassified into 8 clusters (**Figure 2G**). Mitochondrial gene expression was low across most clusters, with the exception of cluster 7, which exhibited relatively higher mitochondrial and ribosomal gene proportions (**Supplementary Figure 2C**). Differential gene expression analysis further showed limited transcriptional divergence among neutrophil clusters except cluster 3, 4, and 5 (**Figure 2H**). Analysis of neutrophil cluster distributions across experimental groups revealed that clusters 3 and 4 were significantly enriched in hyperoxic lungs at Day 7 and Day 14 (**Supplementary Figure 4**). Bar plot confirmed that these increases were time dependent, with minimal differences at Day 3 but marked enrichment at later time points. These results suggest that neutrophil cluster 3 and 4 are closely associated with hyperoxia-induced BPD and progressively expand during disease progression. In addition, the proportion of cluster 1 is higher in normoxia and increases with age, which should be paid more attentions (**Figure 2I**).

In contrast, dendritic cells were subdivided into 4 clusters, but no hyperoxia- or normoxia-specific enrichment was observed. UMAP visualization and differential gene expression analyses demonstrated clear transcriptional distinctions among DC clusters (**Supplementary Figures 3A, B**). However, neither cell number nor proportional analyses revealed significant group-specific differences across time points. In particular, the proportion of cluster 1 showed a significant increase in the normoxia at Day 7. (**Supplementary Figures 3C, D**).

Collectively, these analyses identified several hyperoxia-associated myeloid cell clusters, including monocyte cluster 3, macrophage cluster 1, 3, and 7, and neutrophil cluster 3 and 4. To determine whether the six myeloid cell clusters identified in the single-cell transcriptomic analysis of BPD mouse lungs are also present in human myeloid cells, we collected peripheral blood transcriptomic datasets of preterm infants with BPD from the GEO database. This dataset included blood samples from both healthy preterm infants and preterm infants diagnosed with BPD, with transcriptomic profiles obtained at three time points: cord blood, postnatal day 14, and postnatal day 28. Differential expression analysis was first performed to identify genes specifically upregulated in each myeloid cell cluster. Subsequently, gene set variation analysis (GSVA) was applied to calculate enrichment scores for the six myeloid cell–specific gene signatures in human peripheral blood transcriptomic data. These enrichment scores were used to estimate the relative abundance of each myeloid cluster across different clinical groups. Analysis of human peripheral blood transcriptomes revealed that monocyte cluster 3 exhibited an increasing trend in the BPD group at postnatal day 14 and was significantly enriched at postnatal day 28 (**Supplementary Figure 5A**). Macrophage cluster 1, 3, and 7 showed statistically significant enrichment only at postnatal day 28 (**Supplementary Figure 5**B to D). Similarly, neutrophil cluster 3 and 4 were significantly enriched in the BPD group at postnatal day 28 (**Supplementary Figures 5E, F**). These findings suggest that although these six myeloid cell clusters may be present at relatively low levels during the early stages of BPD, their associated gene signatures become progressively upregulated as the disease advances.

### Validation of BPD-associated myeloid cell subpopulations in peripheral blood of preterm infants with BPD

**Figure 3:**
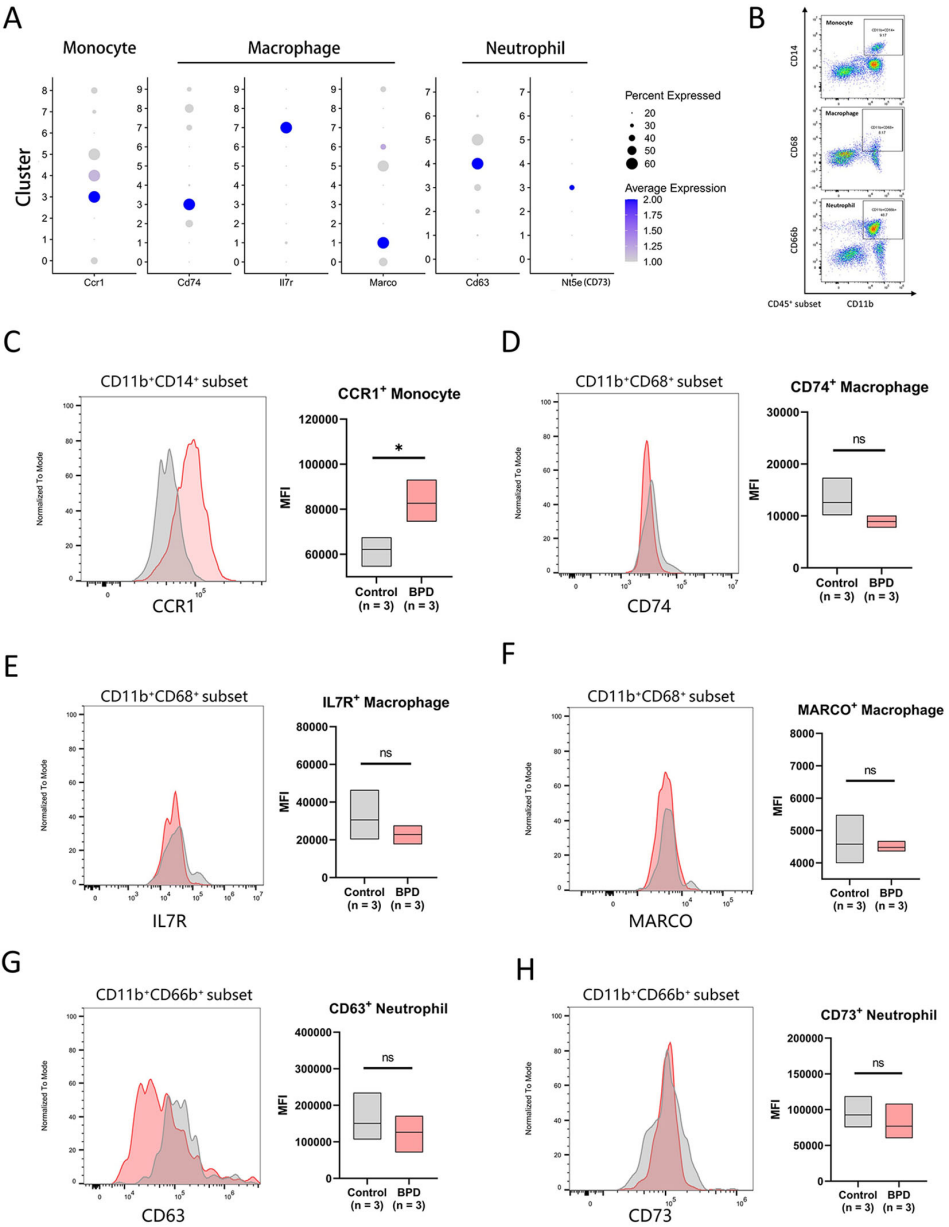
Identified of the BPD-related myeloid subpopulations in human peripheral blood. **(A)** Dot plot showing expression of Ccr1 in all monocyte clusters, expression of Cd74, Il7r, Marco in all macrophage clusters, expression of Cd63, Nt5e (CD73) in all neutrophil clusters. **(B)** Flow cytometry analysis strategy of monocyte, macrophage, neutrophil in human peripheral blood. **(C-H)** Flow cytometry analysis comparing proportions of CCR1^+^ monocyte **(C)**, CD74^+^ macrophage **(D)**, IL7R^+^ macrophage **(E)**, MARCO^+^ macrophage **(F)**, CD63^+^ neutrophil **(G)**, CD73^+^ neutrophil **(H)** between normal and BPD infants, summarized in the box plot. *P < 0.05, ns, not significant.

Despite the GSVA-based estimation indicating enrichment of these six myeloid clusters during later stages of BPD, this approach does not definitively confirm the physical presence of the corresponding cell clusters, but rather reflects increased expression of representative gene signatures. To further validate the relationship between these clusters and BPD, we collected peripheral blood samples from three healthy preterm infants and three preterm infants with BPD at postnatal dat 30, and performed flow cytometric analysis. Prior to flow cytometry, we examined genes encoding membrane protein among the upregulated gene sets for each cluster and selected the most prominently upregulated membrane proteins as specific markers (**Figure 3A**).

We distinguished monocytes, macrophages, neutrophils based on the markers previously reported through flow cytometry analysis (**Figure 3B**). Within the CD11b^+^CD14^+^ monocyte population, the representative surface marker CCR1 of monocyte cluster 3 was significantly upregulated in the peripheral blood of preterm infants with BPD. Analysis of mean fluorescence intensity across all samples confirmed that this increase was statistically significant (**Figure 3C**). In contrast, analysis of other myeloid subpopulations did not fully recapitulate the trends observed in transcriptomic analyses. Within the CD11b^+^CD68^+^ macrophage population, expression of surface marker CD74 of cluster 3, surface marker IL7R of cluster 7, and surface marker MARCO of cluster 1 did not show significant increases in the BPD group and instead exhibited a downward trend in mean fluorescence intensity (**Figures 3D–F**). Similarly, within the CD11b^+^CD66b^+^ neutrophil population, surface marker CD63 of cluster 4 and surface marker CD73 of cluster 3 did not differ significantly between BPD and control groups (**Figures 3G, H**). These results highlight the differences between transcriptomic changes and protein-level expression.

### Differential expression analysis of BPD-associated CCR1^+^ monocytes

Across transcriptomic analyses, the proportion of CCR1^+^ monocytes was progressively increased during lung injury in BPD mouse models and was closely associated with BPD progression in the peripheral blood of preterm infants. Importantly, flow cytometric analysis of human peripheral blood monocytes further demonstrated a significant enhancement of CCR1 expression in preterm infants with BPD. Together, these findings indicate that CCR1^+^ monocytes exhibit a strong association with BPD pathogenesis. To further investigate the downstream signaling and molecular mechanisms of CCR1^+^ monocytes, we analyzed the differentially expressed genes of monocyte cluster 3 and validated these findings using transcriptomic and serum data from human preterm infants.

**Figure 4:**
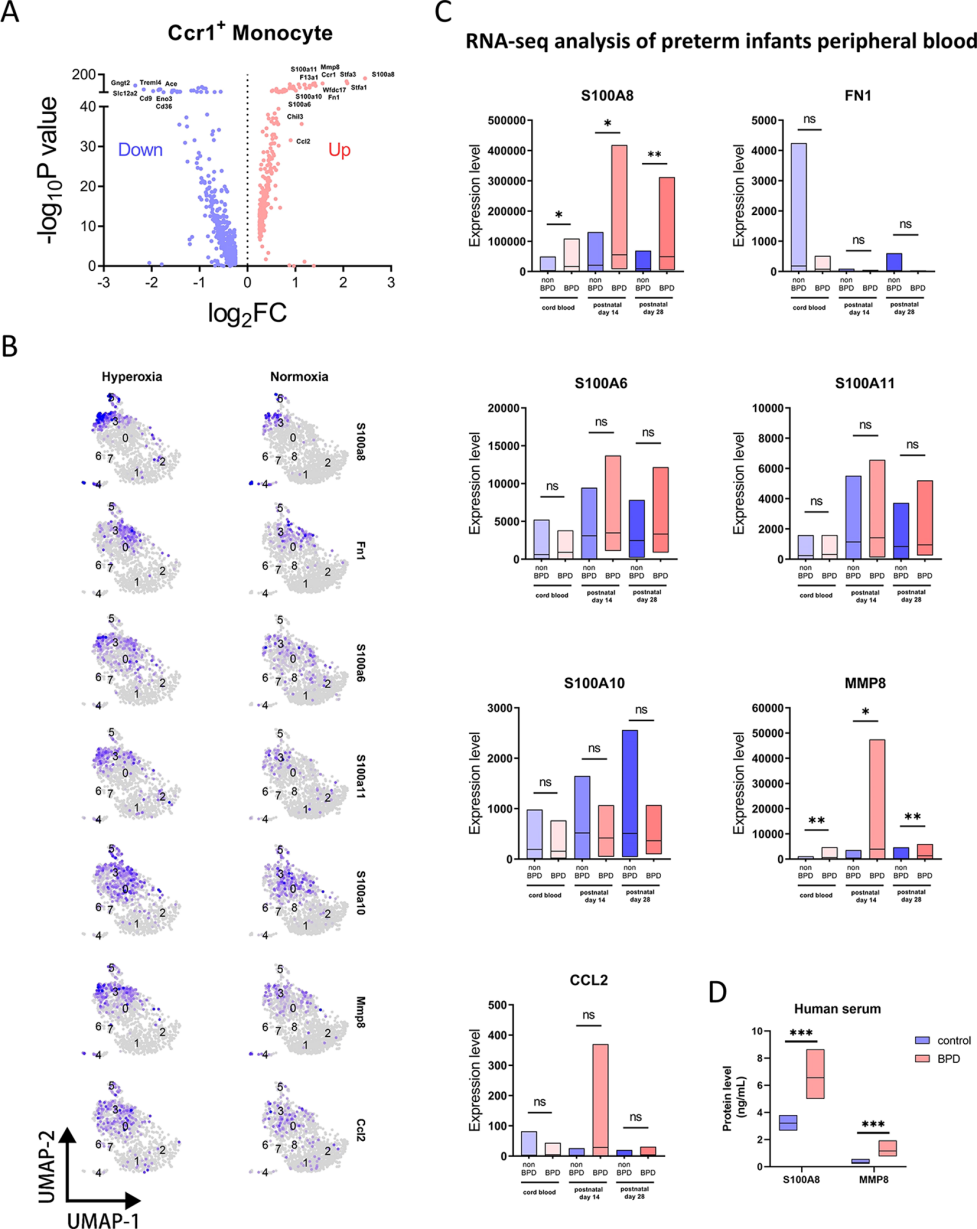
Differential expression analysis of CCR1^+^ monocytes. **(A)** Volcano plot showing differentially expressed genes of Ccr1^+^ monocyte in sc-RNAseq data. **(B)** UMAP plots displaying the distribution of upregulated genes of Ccr1^+^ monocyte in all clusters between hyperoxia and normoxia group. **(C)** Box plots comparing expression of upregulated genes of Ccr1^+^ monocyte between non-BPD and BPD group across birth time in RNAseq data of preterm infants peripheral blood. **(D)** Box plots comparing human serum protein expression levels of S100A8 and MMP8 between control and BPD group by ELISA assays. *P < 0.05, **P < 0.01, ***P < 0.001, ns, not significant.

Differential gene expression analysis revealed that Ccr1^+^ monocytes exhibited upregulation of multiple pro-inflammatory factors (**Figure 4A**), including S100a8, Fn1, S100a6, S100a11, S100a10, Mmp8, and Ccl2. Members of the S100 protein family are key mediators in the initiation and maintenance of inflammatory responses, acting both as downstream responders and amplifiers of inflammatory signaling. Myeloid cell–derived S100A6, S100A8, S100A10, and S100A11 have been implicated in the progression of multiple inflammatory diseases (21–23). Fibronectin (FN1) exists in both soluble and insoluble forms, and the insoluble form secreted by monocytes constitutes an important component of the extracellular matrix, where it interacts with integrin receptors to promote tissue inflammation and fibrosis (24). MMP8, a member of the matrix metalloproteinase family, facilitates inflammatory responses by proteolytically remodeling extracellular matrix components and other extracellular proteins (25). CCL2, also known as monocyte chemoattractant protein-1 (MCP-1), is both a potent inflammatory mediator and a major chemokine for monocyte recruitment, and plays a critical role in inflammatory diseases such as rheumatoid arthritis, inflammatory bowel disease, and respiratory tract infections (26). We next compared the expression levels and spatial distribution of these factors between normoxic and hyperoxic conditions in UMAP projections (**Figure 4B**). These inflammatory mediators were predominantly expressed in Ccr1^+^ monocytes and were upregulated in hyperoxia group.

We further validated the association of these factors with BPD using human peripheral blood transcriptomic data. Comparative analyses across cord blood, postnatal day 14, and postnatal day 28 revealed that only S100A8 and MMP8 were significantly upregulated in the peripheral blood of preterm infants with BPD at all times. It is worth noting that the expression levels of S100A8 and MMP8 showed a varying degree of decrease at postnatal day 28. In contrast, FN1, S100A6, S100A11, S100A10, and CCL2 did not exhibit expression patterns consistent with prior analyses (**Figure 4C**). To further confirm the association of S100A8 and MMP8 with BPD at the protein level, we measured their concentrations in peripheral serum samples from healthy and BPD preterm infants at postnatal dat 30. Both S100A8 and MMP8 protein levels were significantly elevated in the serum of preterm infants with BPD, with S100A8 showing a higher expression than MMP8, consistent with transcriptomic findings (**Figure 4D**). Collectively, these data suggest that CCR1^+^ monocytes contribute to BPD-associated inflammatory responses through the secretion of downstream inflammatory mediators S100A8 and MMP8.

### Validation of CCR1^+^ monocytes in the mouse lungs with BPD

**Figure 5:**
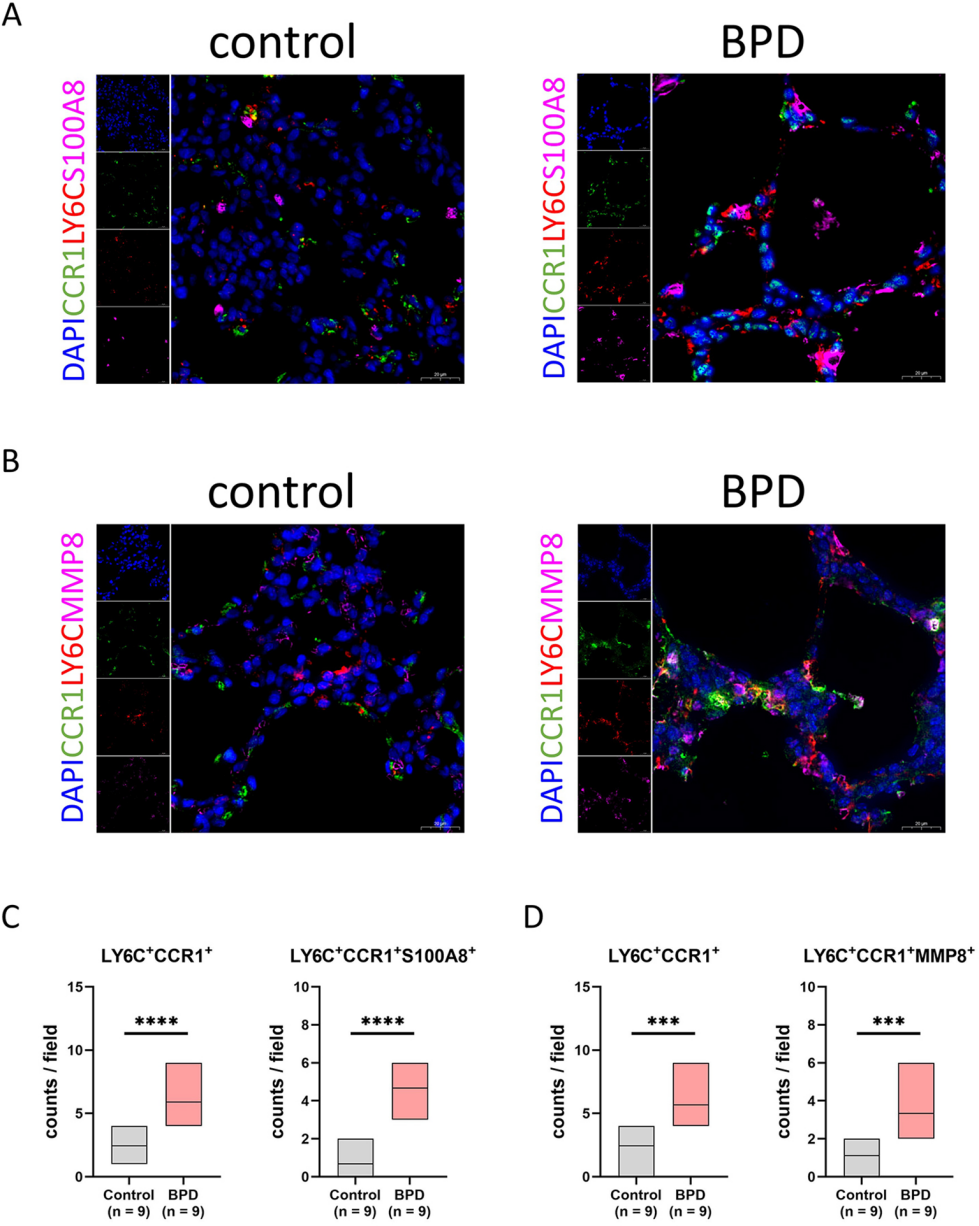
Verification of CCR1^+^ monocytes in hyperoxia-impaired murine lung. **(A, B)** The immunofluorescence shoots showing colocalization of CCR1, LY6C, and S100A8 in mouse normal and BPD lung, summarized in the box plot. **(C, D)** The immunofluorescence shoots showing colocalization of CCR1, LY6C, and MMP8 in mouse normal and BPD lung, summarized in the box plot. Scale bars indicate 20 μm. ***P < 0.001, ****P < 0.0001.

Although increased proportions of CCR1^+^ monocytes and elevated serum levels of S100A8 and MMP8 were observed in the peripheral blood of preterm infants with BPD, it remained unclear whether CCR1^+^ monocytes and their downstream effectors are present within lung tissue and whether they are associated with BPD progression. To address this question, it was necessary to validate our peripheral blood findings within the pulmonary microenvironment. However, due to the limited availability of human lung tissue samples from preterm infants with BPD, we performed immunofluorescence validation using lung tissues from a hyperoxia-induced BPD mouse model. DAPI (blue) was used to label nuclear DNA in all cells, LY6C (red) to identify monocytes within lung tissue, CCR1 (green) to label the CCR1 membrane protein, and S100A8 or MMP8 (pink) to detect intracellular or secreted S100A8 and MMP8 proteins. Lung tissues from mice exposed to hyperoxia (85% O_2_) or normoxia (21% O_2_) were sectioned and stained to compare the microenvironmental and structural differences between hyperoxia-induced BPD lungs and normoxic lungs (**Figures 5A–D**). Compared with normoxic lungs, hyperoxia-exposed lungs exhibited markedly reduced alveolar density and severe disruption of lung architecture. Quantitative analysis revealed a significant increase in LY6C^+^CCR1^+^ monocytes in BPD lungs. Moreover, systematic counting across multiple microscopic fields demonstrated that the numbers of LY6C^+^CCR1^+^ monocytes expressing or secreting S100A8 or MMP8 were also significantly elevated in BPD lung tissues. These findings indicate that CCR1^+^ monocytes and their downstream inflammatory S100A8 and MMP8 are not restricted to the peripheral circulation but are enriched within the pulmonary microenvironment, where they likely exert pro-inflammatory effects that promote local inflammation and contribute to BPD progression.

### NF-κB and AKT signaling pathways differentially regulate S100A8 and MMP8 expression in CCR1^+^ monocytes

**Figure 6:**
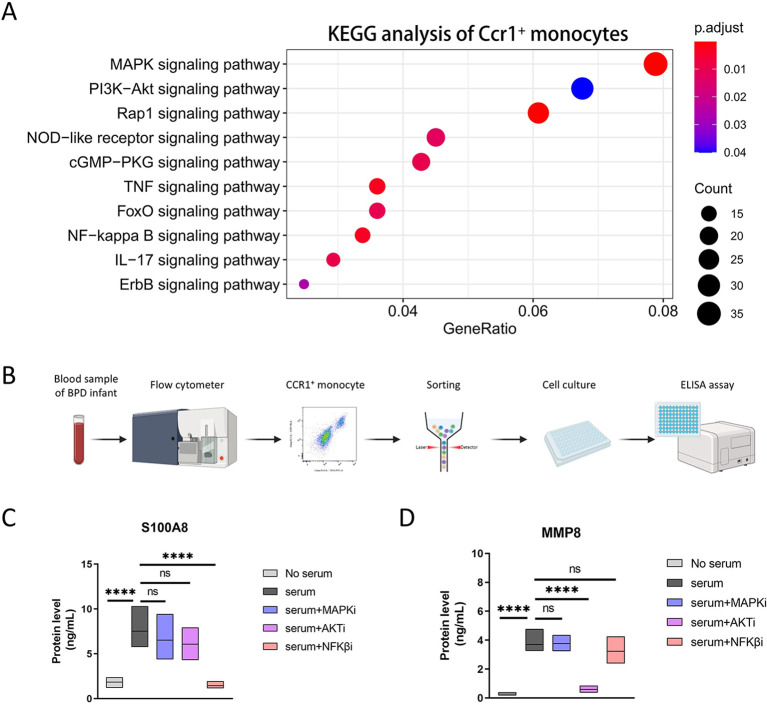
CCR1^+^ monocytes secrete S100A8 and MMP8 through NFκB and AKT pathways respectively. **(A)** Dot plots showing KEGG analysis of differentially expressed genes of Ccr1^+^ monocytes. **(B)** Strategy for isolation of CCR1^+^ monocytes from human BPD premature infants. **(C, D)** The box plots examining S100A8 **(C)** and MMP8 **(D)** protein levels of CCR1^+^ monocytes after inhibiting MAPK, NFκB or AKT pathway. ****P < 0.0001, ns, not significant.

To further elucidate the molecular mechanisms by which CCR1^+^ monocytes regulate S100A8 and MMP8 expression, we performed KEGG pathway enrichment analysis on the CCR1^+^ monocyte cluster identified in the single-cell transcriptomic dataset. In addition to enrichment of TNF- and IL-17–related inflammatory pathways, we observed significant activation of the MAPK, AKT, and NF-κB signaling pathways in CCR1^+^ monocytes (**Figure 6A**). The strong enrichment of these canonical upstream signaling pathways suggested that they may regulate the expression and secretion of S100A8 and MMP8.

To investigate the upstream regulatory mechanisms of S100A8 and MMP8 in human CCR1^+^ monocytes, we collected peripheral blood samples from preterm infants with BPD. CCR1^+^ monocytes were isolated by flow cytometric sorting of CD45^+^CD11b^+^CD14^+^CCR1^+^ cells and cultured *in vitro* (**Figure 6B**). These cells were stimulated with autologous serum from BPD infants, while cells cultured without serum stimulation served as negative controls. To dissect pathway-specific regulation, CCR1^+^ monocytes were treated with the MAPK inhibitor SCH772984 (27), the NF-κB inhibitor BAY11-7082 (28), or the AKT inhibitor MK-2206 (2HCl) (29) under serum-stimulated conditions. The levels of S100A8 and MMP8 in culture supernatants were quantified using ELISA. The results demonstrated that serum from preterm infants with BPD significantly enhanced the expression and secretion of both S100A8 and MMP8 in CCR1^+^ monocytes, indicating the presence of circulating upstream activators in the serum. Pharmacological inhibition experiments further revealed that blockade of the NF-κB pathway markedly suppressed S100A8 secretion, whereas inhibition of the MAPK or AKT pathways resulted in a non-significant downward trend in S100A8 levels. In contrast, MMP8 expression and secretion were predominantly regulated by the AKT pathway, as treatment with the AKT inhibitor significantly reduced serum-induced MMP8 levels to those observed in the negative control group (**Figures 6C, D**).

Collectively, these findings demonstrate that CCR1^+^ monocytes promote BPD progression by activating distinct signaling pathways: the NF-κB pathway drives the production and release of S100A8, whereas the AKT pathway primarily regulates MMP8 expression and secretion. These pathway-specific mechanisms further highlight the pathogenic role of CCR1^+^ monocytes in BPD-associated inflammation.

## Discussion

In this study, we identified myeloid cell subpopulations associated with BPD by analyzing transcriptomic data of human and mouse BPD tissues. Flow cytometry analysis of peripheral blood cells from BPD preterm infants further confirmed association of CCR1^+^ monocytes with disease development. Subsequently, *in vitro* experiments were conducted to elucidate the molecular mechanisms by which CCR1^+^ monocytes participate in BPD-associated inflammatory responses. Our results demonstrated that CCR1^+^ monocytes promote the production and release of the inflammatory proteins S100A8 and MMP8 via activation of the NF-κB and AKT signaling pathways, respectively. These findings were further validated in peripheral blood samples from preterm infants with BPD and lung tissues from a murine BPD model.

S100A8 is an important calcium- and zinc-binding protein that forms a stable heterodimer with S100A9, known as calprotectin. The S100A8/9 complex not only recruits and activates inflammatory cells such as neutrophils, monocytes, and macrophages at sites of inflammation, but also amplifies downstream inflammatory responses by promoting the release of pro-inflammatory cytokines (30). Consistent with our findings, previous studies have reported significantly elevated serum levels of S100A8 in preterm infants with BPD (31). Moreover, increased S100A8 expression has been observed in lung tissues of hyperoxia-induced murine BPD models, with CXCL4 proposed as an upstream regulator of macrophage-derived S100A8 (32). These observations raise the possibility that, within the BPD lung microenvironment, CXCL4 may act as a chemokine to recruit CCR1^+^ monocytes to the lung and promote S100A8 production, thereby exacerbating local inflammatory signaling. This hypothesis warrants further investigation.

MMP8 plays a central role in the degradation of extracellular matrix collagen and is also capable of processing non-matrix substrates, including cytokines such as CXCL5, CXCL8, CXCL10, and CCL2, thereby modulating inflammatory signaling pathways. Aberrant overexpression and excessive activation of MMP8 have been implicated in the progression of various diseases, including rheumatoid arthritis, asthma, periodontitis, atherosclerosis, and cancer (33, 34). MMP8 has traditionally been considered to be predominantly released by neutrophils through degranulation. However, our study reveals that under the pathological conditions of BPD lung tissue, CCR1^+^ monocytes also represent a significant source of MMP8. The extracellular matrix–degrading function of MMP8 may be closely associated with the infiltration of CCR1^+^ monocytes into lung tissue. Notably, we did not further assess the activation status of MMP8 or the levels of upstream enzymes involved in its activation, such as MMP3, MMP7, and MMP10 (35), which merits more comprehensive and systematic investigation. In addition, recent studies have linked MMP8 to pulmonary arterial hypertension, a major cause of mortality in preterm infants with moderate to severe BPD, and pharmacological targeting of MMP8 has been shown to alleviate pulmonary hypertension and improve cardiac function (36).

In addition to CCR1^+^ monocytes, transcriptomic analyses also identified several other macrophage and neutrophil subpopulations. Although the findings from peripheral blood analyses in human BPD infants were not fully consistent with transcriptomic results, this discrepancy may be attributable to the selection of suboptimal markers. Previous studies have reported that activated peripheral blood neutrophils can secrete CD63^+^CD66b^+^ exosomes, which degrade the extracellular matrix via exosome-associated neutrophil elastase (NE), ultimately contributing to lung extracellular matrix damage and promoting the development of BPD (37). Based on the reported high expression of CD63 in these exosomes, it is reasonable to further investigate the role of CD63^+^ neutrophil subpopulations in BPD. Such studies should include the identification of more appropriate markers to validate this subset and exploration of whether CD63 is shed together with exosomes following its expression.

Several limitations of this study should be acknowledged. Although we confirmed the presence and molecular mechanism of CCR1^+^ monocytes, the interactions with other cell types remain to be fully characterized. Furthermore, we verified the expression of S100A8 and MMP8 in CCR1^+^ monocytes, but the contribution of these CCR1^+^ monocyte–derived factors to BPD pathogenesis remains unclear. Addressing these questions will require more comprehensive approaches, including genetically modified animal models, advanced molecular biology techniques, and large-scale database analysis.

In conclusion, our study delineates the roles and heterogeneity of myeloid cells in the development of BPD in preterm infants, identifies distinct myeloid cell subsets associated with different stages of disease progression, and confirms the association between a specific monocyte subset and its downstream effectors with BPD using clinical samples and animal models. By integrating transcriptomic analyses with *in vitro* functional experiments, we further elucidate the underlying molecular mechanisms. These findings may facilitate the development of diagnostic strategies for preterm infants with BPD and provide new perspectives for the identification of therapeutic targets.

## Data Availability

Publicly available datasets were analyzed in this study. This data can be found here: https://www.ncbi.nlm.nih.gov/geo/query/acc.cgi?acc= GSE151974, GSE151974, GEO, NCBI. https://www.ncbi.nlm.nih.gov/geo/query/acc.cgi?acc= GSE220135, GSE220135, GEO, NCBI.
