# Supplementary material for: CCR1+ monocytes facilitating bronchopulmonary dysplasia through regulation of S100A8 and MMP8
Source: Front Immunol. 2026 May 1;17:1809178. doi: 10.3389/fimmu.2026.1809178 (PMC13175868; doi:10.3389/fimmu.2026.1809178)
Supplement: Supplementary file 1 [file Table1.docx]

**Supplementary Figure 1. Differential gene analysis of all cell types.** Heatmap showing representative marker genes of all cell types.

**Supplementary Figure 2. Analysis of mitochondrial and ribosomal genes. (A-D)** Violin plots showing proportions of mitochondrial and ribosomal genes in monocytes (A), macrophages (B), neutrophils (C), and DCs (D).

**Supplementary Figure 3. Sc-RNAseq analysis of DCs. (A)** UMAP plot displaying cell clustering of DCs. **(B)** Heatmap showing representative marker genes of all DC clusters. **(C)** UMAP plots comparing clustering of all DC clusters in normoxia and hyperoxia groups on day 3, day 7, or day 14. **(D)** Histogram comparing proportions of all DC clusters in normoxia and hyperoxia groups on day 3, day 7, or day 14.

**Supplementary Figure 4. Clustering analysis of monocytes, macrophages, and neutrophils.** UMAP plots comparing clustering of all monocyte, macrophage, and neutrophil clusters in normoxia and hyperoxia groups on day 3, day 7, or day 14.

**Supplementary Figure 5. Verification of mouse BPD-associated clusters in premature infant blood with BPD. (A-F)** Infiltration scores of monocyte cluster 3 (A), macrophage cluster 1 (B), macrophage cluster 3 (C) , macrophage cluster 7 (D), neutrophil cluster 3 (E), neutrophil cluster 4 (F) in premature infant blood with BPD, predicted by GSVA arithmetic. Ns, not significant, *P < 0.05, **P < 0.01, ***P < 0.001.
