# Supplementary figures and images for: CCR1+ monocytes facilitating bronchopulmonary dysplasia through regulation of S100A8 and MMP8
Source: Front Immunol. 2026 May 1;17:1809178. doi: 10.3389/fimmu.2026.1809178 (PMC13175868; doi:10.3389/fimmu.2026.1809178)

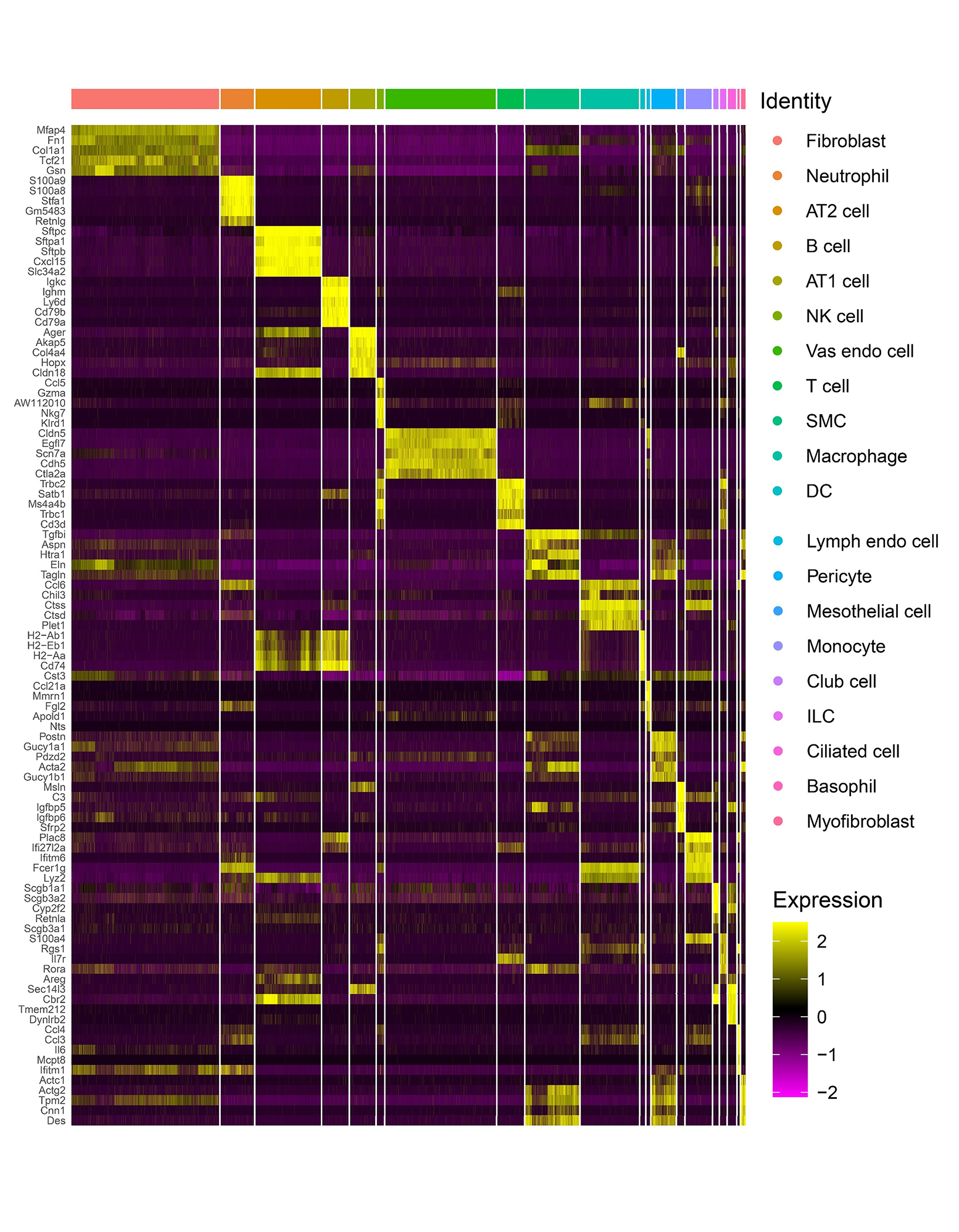

Supplement: Supplementary file 2 [file Image1.jpeg]

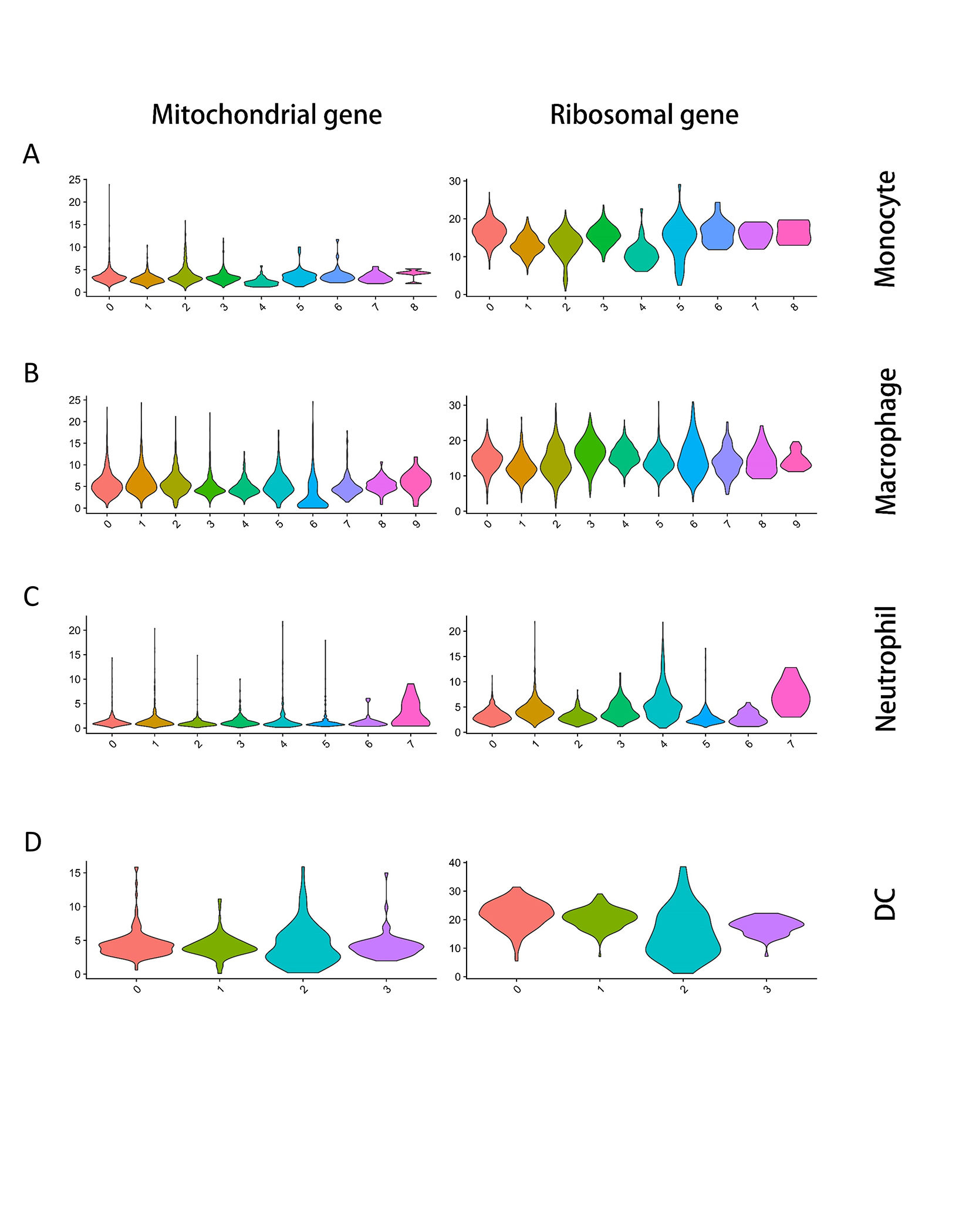

Supplement: Supplementary file 3 [file Image2.jpeg]

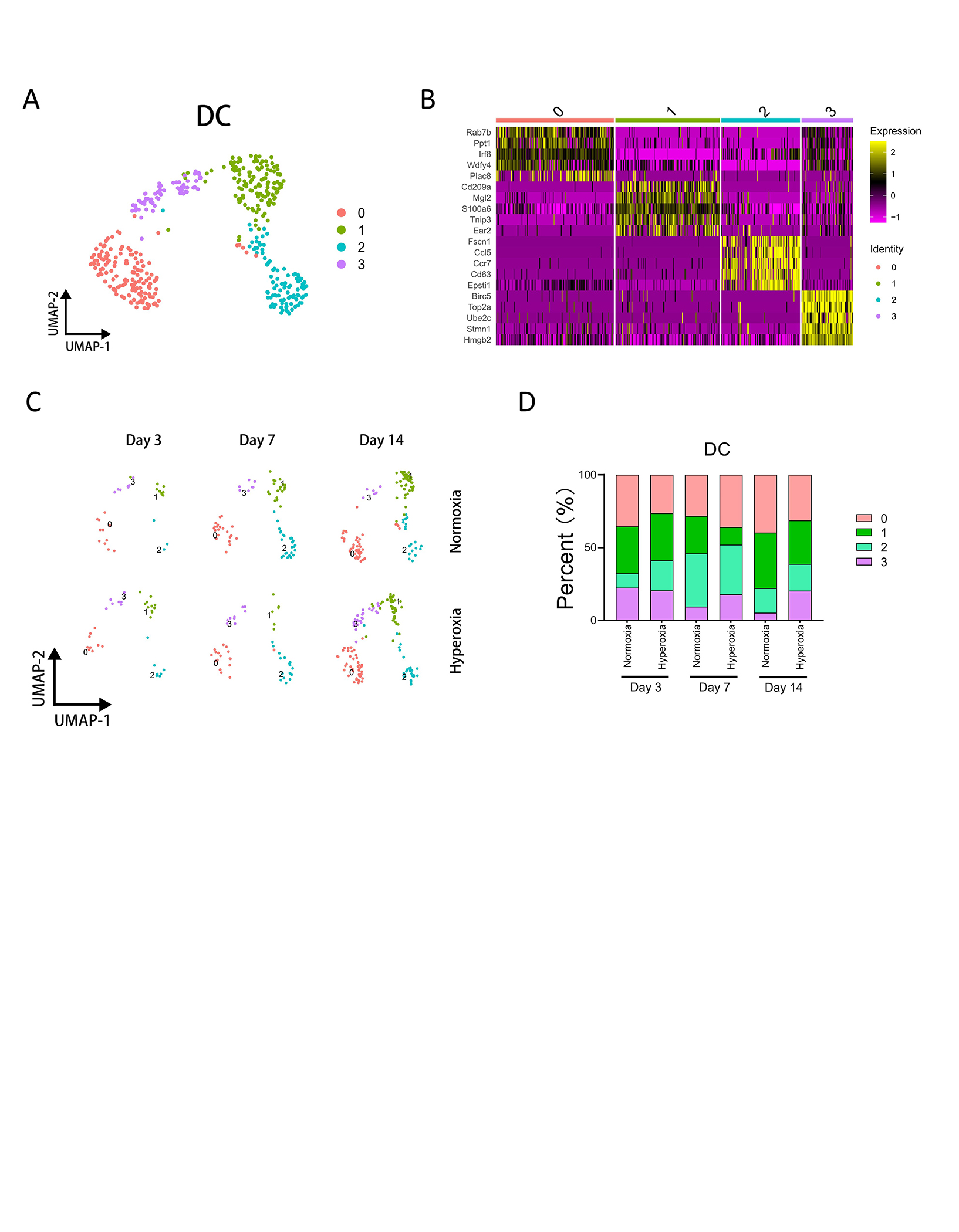

Supplement: Supplementary file 4 [file Image3.jpeg]

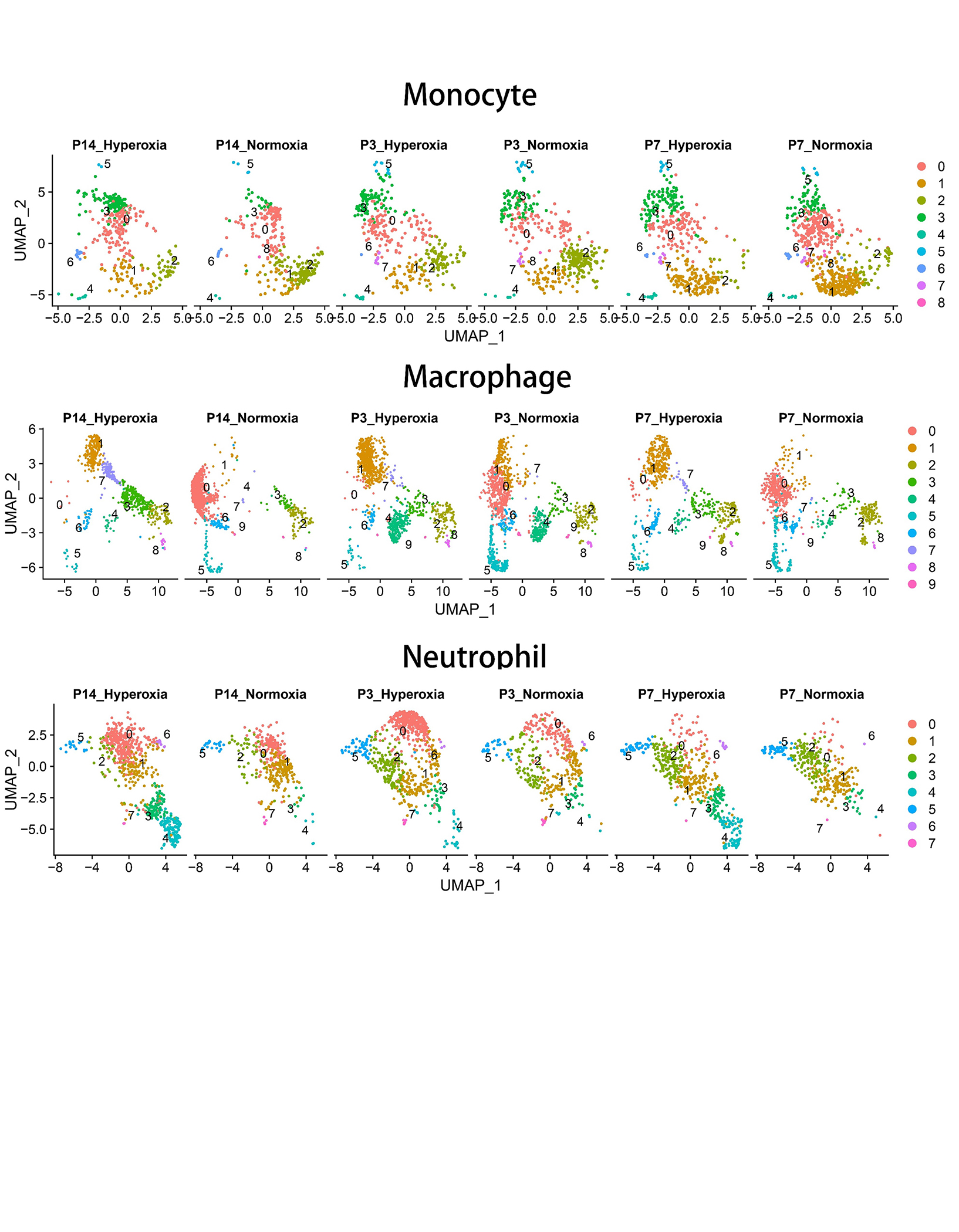

Supplement: Supplementary file 5 [file Image4.jpeg]

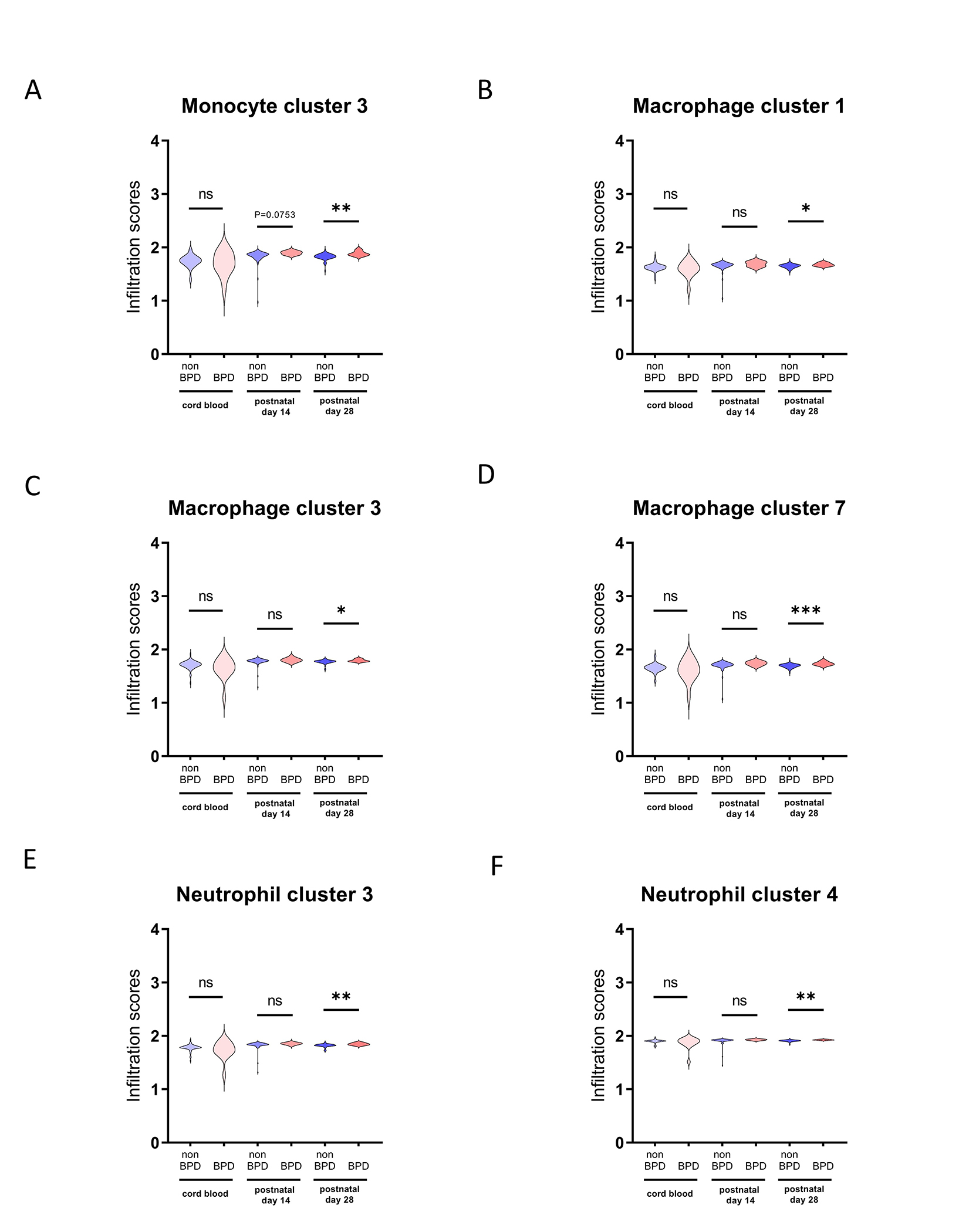

Supplement: Supplementary file 6 [file Image5.jpeg]
